# Markerless Image Alignment Method for Pressure-Sensitive Paint Image

**DOI:** 10.3390/s22020453

**Published:** 2022-01-07

**Authors:** Kyosuke Suzuki, Tomoki Inoue, Takayuki Nagata, Miku Kasai, Taku Nonomura, Yu Matsuda

**Affiliations:** 1 Department of Modern Mechanical Engineering, Waseda University, 3-4-1 Ookubo, Shinjuku-ku, Tokyo 169-8555, Japan; kyosuke.tree_212@fuji.waseda.jp (K.S.); deseiko@ruri.waseda.jp (T.I.); 2 Department of Aerospace Engineering, Tohoku University, 6-6-01 Aoba, Arakaki, Aoba-ku, Sendai 980-8579, Miyagi, Japan; nagata@tohoku.ac.jp (T.N.); miku.kasai.p8@dc.tohoku.ac.jp (M.K.); nonomura@tohoku.ac.jp (T.N.); 3 Japan Science and Technology Agency (JST), PRESTO, 4-1-8 Honcho, Kawaguchi 332-0012, Saitama, Japan

**Keywords:** pressure-sensitive paint, image alignment, feature point detection, flow measurement

## Abstract

We propose a markerless image alignment method for pressure-sensitive paint measurement data replacing the time-consuming conventional alignment method in which the black markers are placed on the model and are detected manually. In the proposed method, feature points are detected by a boundary detection method, in which the PSP boundary is detected using the Moore-Neighbor tracing algorithm. The performance of the proposed method is compared with the conventional method based on black markers, the difference of Gaussian (DoG) detector, and the Hessian corner detector. The results by the proposed method and the DoG detector are equivalent to each other. On the other hand, the performances of the image alignment using the black marker and the Hessian corner detector are slightly worse compared with the DoG and the proposed method. The computational cost of the proposed method is half of that of the DoG method. The proposed method is a promising for the image alignment in the PSP application in the viewpoint of the alignment precision and computational cost.

## 1. Introduction

The pressure-sensitive paint (PSP) technique is an optical flow diagnosis method for measuring pressure on a model to which a PSP coating is applied [1,2,3,4]. The PSP technique has received much attention, as it enables non-intrusively measuring pressure distribution. Indeed, the PSP technique has been applied to various fluid dynamic experiments: wind tunnel testing [5,6,7,8,9], rotating machineries [10,11,12], and micro devices [13,14,15,16,17]. In the PSP technique, pressure is measured by the variation of the luminescence intensity from the PSP coating with a CCD/CMOS camera [2,3]. To improve the measurement accuracy of the PSP technique, a temperature compensation method is intensively investigated [18,19,20,21,22,23,24,25]. In addition, there are many studies on measurement system [26,27] and post-processing [28,29,30,31] for the noise reduction in the PSP image. On the other hand, there is less research on an image alignment method. The alignment of the reference and the run images is an important procedure in the post-processing of PSP data, because the misalignment of the images leads to large measurement error. In conventional techniques, black markers placed on a model surface are used to image alignment [32,33]. Sant et al. [32] proposed an automatic method that detects black markers and links those of the reference and the run images. The linking method is based on the distance between the markers, and the method requires an iterative calculation. Since the positions of the markers in both reference and run images are detected and linked manually in the method proposed by Fujimatsu et al. [33], the method is time consuming for practical applications. The advantage of the method proposed by Fujimatsu et al. also uses corners of a model for the image alignment to improve the alignment accuracy. In general, since the positions of black markers are heuristically determined, the performance of the image alignment will vary by the researchers. In these conventional methods using black markers, pressures at the markers cannot be measured.

In this study, we focus on features of images such as corners, edges, and contours [34]. We propose an image alignment method using features and its feature values, while the above-mentioned methods do not use feature values; that is, the existing method only uses the information of the marker positions. The use of feature values, characterizing the neighborhood of the features [35], enables us to easily link the features between the reference and the run images and to align the images without using black markers. Although there are many studies on image alignment/registration in various fields such as computer vision and medical image fields [34,35], these methods are not directly applicable to the PSP images. This is because the images obtained in the PSP measurement usually consist of white solid color (luminescence from PSP) and black solid color (background) parts with few features. Kuzub et al. [36] proposed the method using feature points. Since the features on the PSP such as small scratches and defects are usually affected by the illumination, the mismatching of the features between the reference and the run images will occur due to the change of the illumination condition. Then, Kuzub et al. also used the black markers. In this study, we propose a markerless image alignment method based on a boundary detection method. The performance of the proposed method is compared with the conventional method based on black markers, the difference of Gaussian (DoG) detector, and the Hessian corner detector.

## 2. Lifetime-Based PSP Measurements

A PSP coating consists of phosphorescence dye and binder and is applied to a model surface. When the dye is photoexcited, the phosphorescence is emitted from the dye. The intensity/lifetime of the phosphorescence depends on pressure due to oxygen quenching. Therefore, the pressure on the model can be measured by detecting the variation of the intensity/lifetime; there are two approaches for measuring pressure distribution: the intensity-based method and lifetime-based one [2,3]. In the intensity-based method, the change in position of the model will induce the error due to the change in the illumination distribution. As a demonstration of the image alignment methods, we used a lifetime-based method known as the two-gated lifetime method [37,38,39,40] in this study. Two images are captured in the two-gated lifetime method: Gate 1 image is captured during the excitation of PSP, and Gate 2 one is captured during the decay of the luminescence of PSP after excitation light is off. Then, the image ratio R is calculated as

(1) $$R=\frac{I_{\mathrm{Gate}1}}{I_{\mathrm{Gate}2}}$$

where $I_{\mathrm{Gate}1}$ and $I_{\mathrm{Gate}2}$ are Gate 1 and Gate 2 images, respectively. Theoretically, the image ratio $R_{\mathrm{run}}$ obtained at the run condition relates to pressure. However, it is difficult to obtain pressure distribution from $R_{\mathrm{run}}$ due to the non-uniformity of the lifetime spatial distribution. To compensate for the effect of this non-uniformity, the image ratio $R_{\mathrm{ref}}$ obtained at the reference condition is used. Then, the ratio of $R_{\mathrm{run}}$ and $R_{\mathrm{ref}}$ is used for pressure measurement [41].

(2) $$\frac{R_{\mathrm{run}}}{R_{\mathrm{ref}}}=\underset{i}{\sum }a_{i}{\left(\frac{p_{\mathrm{run}}}{p_{\mathrm{ref}}}\right)}^{i}$$

where $a_{i}$ are calibration constants.

## 3. Image Alignment Method

### 3.1. Feature Point Detection

There are many feature point detection methods detecting rotation invariant and scale-variant feature points. However, the scale of PSP images is usually not changed. As feature point detection methods, we use the following three methods: the difference of Gaussian (DoG) detector, the Hessian corner detector, and the proposed method. It is noted that PSP image is usually a grayscale one. In the DoG detector, the DoG operator, which is an approximation form of the Laplacian of Gaussian (LoG) operator, is applied to image to detect feature point [35,42]. The DoG operator DoG is written as

(3) $$DoG\left(x,y\right)=\frac{1.6\left\{G\left(x,y,1.6\sigma \right)-G\left(x,y,\sigma \right)\right\}}{\sigma^{2}}$$

where G(x,y,σ) is the two-dimensional Gaussian filter at pixel (x,y) of image with the standard deviation σ. After applying the DoG operator, the points with higher values than a threshold are adopted as feature points. In this study, the threshold was determined to detect the feature points near the corners and edges, because the feature points on the model usually lead to mismatching due to the change of the illumination condition between the images.

In the Hessian corner detector, the following Hessian matrix is considered [35,43]:

(4) $$H\left(x,y\right)=\left[\begin{array}{cc} I_{xx}\left(x,y\right) & I_{xy}\left(x,y\right)\\ I_{xy}\left(x,y\right) & I_{yy}\left(x,y\right) \end{array}\right]$$

where I(x,y) indicates the intensity of the image at pixel (x,y) and the subscript indicates the gradient in the x or y direction. For example, $I_{x}\left(x,y\right)=I\left(x+1,y\right)-I\left(x,y\right)$ is the gradient in the x direction, and $I_{y}\left(x,y\right)=I\left(x,y+1\right)-I\left(x,y\right)$ is the y direction gradient. Since the computational cost for Equation (4) is high, we treat the approximation form using box filters [44]. Following Ref. [44], the 9×9 box filters are used to approximately express $I_{xx}$, $I_{yy}$, and $I_{xy}$. The approximated gradients $I_{xx}$, $I_{yy}$, and $I_{xy}$ are denoted as $D_{xx}$, $D_{yy}$, and $D_{xy}$, respectively. Then, the determinant of the Hessian matrix is considered

(5) $$\det\left(H\right)=D_{xx}D_{yy}-{\left(wD_{xy}\right)}^{2}$$

where w is the relative weight of the filter responses and is 0.9 in this study following Ref. [44]. Calculating Equation (4), we adopt the points with higher value of the determinant as feature points.

The proposed method is as follows. The boundary of the PSP/model is traced using the Moore-Neighbor tracing algorithm [45,46]. In the algorithm, the image is binarized, and the outline of the PSP/model is traced in the binary image. A pixel on the boundary of an object has to be specified as a starting point of the tracing. The Harris corner detector [35,47] is used to find the pixel on the boundary (corner) of PSP. The traced outline is varied due to a binarization threshold value; thus, we trace the outlines under six binarized images based on six binarization threshold values. The 0.04, 0.08, 0.12, 0.16, 0.20, and 0.24 are used as binarization threshold values in the PSP image normalized by the maximum intensity value. The obtained six outlines are adopted as feature points. The advantage of this method is its lower computational cost than the conventional method.

### 3.2. Description of Feature Point

As a feature descriptor, a vector of the descriptor with 64 dimensions is used in this study and is calculated from the neighborhood around the feature point following Ref. [44]. For obtaining a rotation-invariant descriptor, the Haar wavelet (size of 4s×4s) responses in the horizontal and vertical axes of the image are calculated for each point within a circular neighborhood of radius 6s around the point of interest, where s is 1.6 following Ref. [44] in this study. Then, the responses are filtered by the Gaussian filter with the standard deviation of 2s. The response angles are calculated as the arctangent of the ratio of the horizontal response to the vertical one. The responses are summed in the sliding angle window of π/3. The orientation of the feature point is determined by the ratio of the summed horizontal response to the vertical one in the region with the maximum summed response. The square region of 20s×20s around the featured point is considered and is rotated in the calculated orientation. Then, the region is divided into 4×4 subregions. The Haar wavelet responses (size of 2s×2s) in the horizontal and vertical axes are calculated from 5×5 regularly sampled points for each subregion. The absolute values of them are also calculated. The Gaussian filter with the standard deviation of 3.3s is also applied following Ref. [44]. These four Haar wavelet responses are obtained for 4×4 subregions; thus, the descriptor with 64 dimensions is obtained for each feature point. In this study, we used OpenSURF code and calculated the descriptor [48].

### 3.3. Matching of Feature Points between Images

Based on the calculated descriptor, the corresponding feature points between the reference and the run image are estimated using the Harris algorithm. We used “matchFeatures” of MATLAB [49], where the pairwise distance between feature vectors are calculated. In the input arguments for “matchFeatures”, the “MaxRatio” was set at 0.9. The default values were used for other arguments. Based on the matched feature points, the geometric transformation matrix is calculated using “estimateGeometricTransform” function [50]. We used “affine” for “transformType” in the function. In this function, the outliers are excluded based on the M-estimator sample consensus [50]. Then, the reference and run images are aligned by applying the transformation matrix.

## 4. Experimental Method

We investigated the performance of the methods using the PSP data of a two-bladed rotor model [51]. In this paper, we briefly introduce the experimental method. The details are provided in Nagata et al. [51]. The chord and the span of the blade were 44.7 mm and 89.4 mm, respectively. The rotor diameter was 303 mm. In this study, we used platinum (II) mesotetra(pentafluorophenyl)porphine (PtTFPP) as a pressure-sensitive dye and poly(isobutyl-co-1,1,1,3,3,3-hexafluoroisopropyl methacrylate) (PHFIPM) as a polymer binder. We painted PSP on only one blade in the following procedure. First, a white polyester film (1080-M10, 3M Japan, Japan) was stuck onto the model. Second, a PHFIPM solution without PtTFPP was applied to it as a buffer layer to prevent a chemical interaction between PtTFPP and the white polyester film. Then, the PSP (PtTFPP/PHFIPM) was applied to it. We also painted six black circular markers on the blade to compare the proposed markerless method with the conventional marker-based one.

The PSP coating was illuminated by an LED device with a central wavelength of 395 nm (IL-106UV LED, HARDsoft Microprocessor Systems, Kraków, Poland). The emission of the PSP coating was captured with repeatedly short exposure time by a CCD camera (PCO1600, PCO, Germany). In this study, we obtained two image ratios with a difference position of the blade, $R_{\mathrm{ref}}$ (before rotation) and $R_{\mathrm{rot}}$ (after rotation), to examine the image alignment methods. It is noted that both image ratio images were obtained under an atmospheric pressure without rotating motion, and the pressure on the blade was uniform; thus, we can evaluate the accuracy of the image alignment by calculating the ratio of the image ratios, $R_{\mathrm{rot}}/R_{\mathrm{ref}}$, which is ideally unity for a well-aligned image.

## 5. Results and Discussion

**Figure 1:**
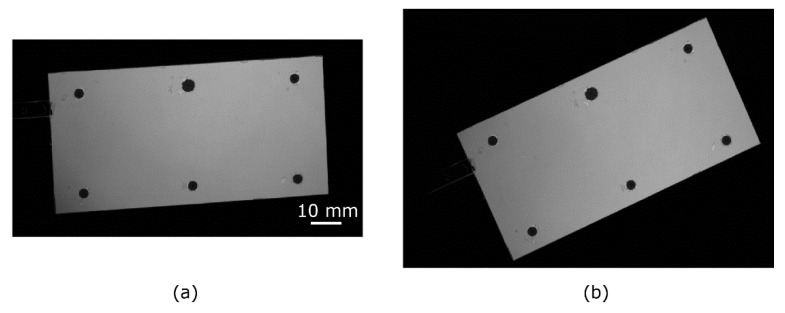
$I_{\mathrm{Gate}1}$ images of blade. (**a**) before rotation, (**b**) rotated 25°. The images are cropped around the blade.

**Figure 2:**
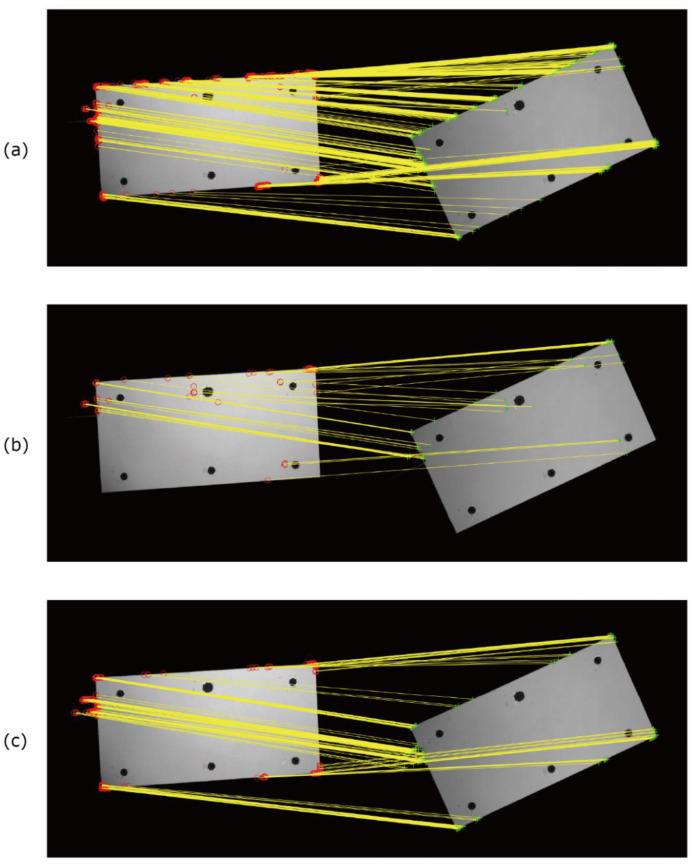
Results of feature point matching for obtained feature points by (**a**) the DoG detector, (**b**) the Hessian corner detector, and (**c**) the proposed method.

**Figure 3:**
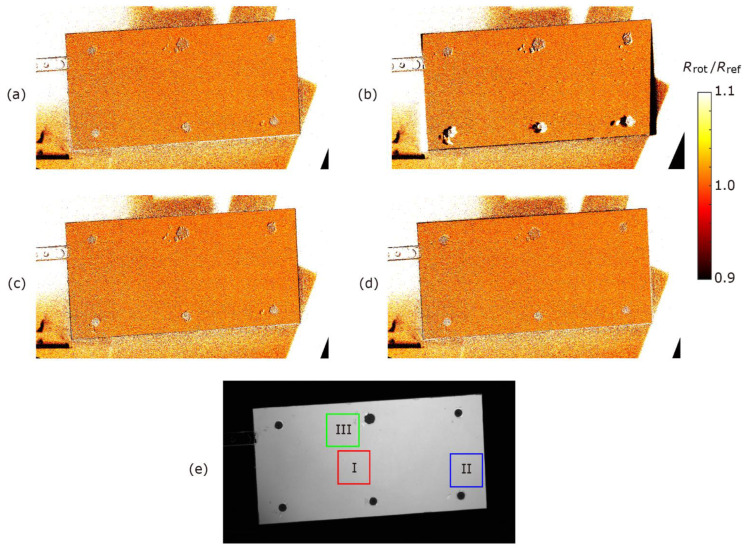
Ratio of image ratios, $R_{\mathrm{rot}}/R_{\mathrm{ref}}$, for aligned images by (**a**) the DoG detector, (**b**) the Hessian corner detector, (**c**) the proposed method, and (**d**) the conventional method using black markers. (**e**) Processing area for Table 1.

Figure 1 shows the $I_{\mathrm{Gate}1}$ images of the blade before rotation and rotated 25°. The images were aligned by the methods introduced in Section 3. As a comparison, the images were aligned by the conventional method using black markers. Figure 2 shows the feature points obtained by each method and the results of feature point matching. The matched points are connected by yellow solid lines. The resultant ratios of the image ratios, $R_{\mathrm{rot}}/R_{\mathrm{ref}}$, are shown in Figure 3. As described in the previous section, this ratio is ideally unity for the well-aligned image. We computed the mean value and standard deviation of $R_{\mathrm{rot}}/R_{\mathrm{ref}}$ for the 100×100 pixels at the center and near edge areas (shown in Figure 3e) of the blade to evaluate the alignment performance. The results are summarized in Table 1. The results shown in here are the best results for each method. The mean values and the standard deviation of $R_{\mathrm{rot}}/R_{\mathrm{ref}}\;$at the center portion of the blade were equal for each method, as shown in Table 1. Due to the shot noise of the camera, the standard deviations of $R_{\mathrm{rot}}/R_{\mathrm{ref}}$ were approximately 0.04 for all cases except for the result of the Hessian corner detector.

Figure 2 and Figure 3 illustrate that the results using the conventional black marker method, the DoG detector, and the proposed method were similar. However, the values of $R_{\mathrm{rot}}/R_{\mathrm{ref}}$ were approximately 0.9 (it is observed as the black line) at the upper portion (leading edge) of the blade for the conventional black marker method. It is considered that the number of the point used for the image alignment was only six, and there were no points for the alignment near the leading edge of the blade in this study. Since the black markers are generally not placed the boundary of the PSP or the edge of the model, the alignment accuracy will be worse near these areas. This indicates that the image alignment accuracy of the conventional black marker method is slightly worse compared with those of the DoG detector and the proposed method. The result obtained by the Hessian corner detector was worse. For example, the values of $R_{\mathrm{rot}}/R_{\mathrm{ref}}$ at the right side and bottom (trailing edge) of the blade were approximately 0.9. Then, the mean and standard deviation of $R_{\mathrm{rot}}/R_{\mathrm{ref}}$ at area II were respectively lower and higher than those of the other methods. This is because there was no matching feature point in the lower-left corner of the blade, as shown in Figure 2b. The luminescent intensity at the lower-left corner area was lower than that at other areas, as shown in Figure 1a, and the feature point was not detected by the Hessian corner detector using a single threshold. As shown in Figure 3 and Table 1, the results obtained by the DoG detector and the proposed method were equivalent. However, the number of matched points was approximately 2.5 times larger in the DoG method than in the proposed method. In other words, the number of matching points of the proposed method was enough to correctly align the images. It is noted that the computational cost of the DoG method (about 2.5 min in a laptop PC of Windows 10 professional with CPU of Intel(R) Core(TM) i7-7500U, Matlab 2020a) was higher than that of the proposed method (approximately 1 min). The proposed method was also confirmed to be successfully applied to the PSP images used in Ref. [31].

## 6. Conclusions

A markerless image alignment method for pressure-sensitive paint (PSP) measurement data was proposed. Since the images are generally aligned using feature points, the performance of image alignment mainly depends on whether the preferred feature points are detected or not. Conventionally, the PSP images are aligned based on black markers placed on a model surface. The positions of the markers are detected manually, and this method is time consuming for practical applications. Since the positions of black markers are heuristically determined, the performance of the image alignment will vary by the researchers. Moreover, pressures at the markers cannot be measured in the method using black markers. Then, we proposed a feature point detection method based on the boundary detection method, in which the PSP/model boundary is detected using the Moore-Neighbor tracing algorithm. The proposed method uses the boundary as feature points instead of the points such as small scratches and defects of the paints, which are affected by the illumination condition. The performance of the image alignment by the proposed method was compared with the conventional method based on black markers, the difference of Gaussian (DoG) detector, and the Hessian corner detector. For this purpose, we measured the PSP images before and after rotation of a rotor blade, to which a PSP was applied. In this study, we used a lifetime-based method to eliminate the effect of illumination distribution before and after rotation. The results obtained by the proposed method and the DoG detector were equivalent to each other. On the other hand, the performances of the image alignment using the black marker and the Hessian corner detector were worse compared with the DoG and the proposed method. In the black marker and the Hessian corner detector, the feature points were not prepared (black marker) or detected (the Hessian corner detector) near or on the edge and corner of the PSP; thus, the alignment accuracy decreased such areas. Now, if we focus on the proposed method and the DoG detector, the number of matched points was approximately 2.5 times larger in the DoG method than in the proposed method. In other words, the number of matching points of the proposed method was enough to correctly align the images and reduces the computational cost. The computational cost of the proposed method is 2/5 of the DoG detector without losing the alignment accuracy. The proposed method will be a powerful markerless alignment method for the PSP data.

## Figures and Tables

**Table 1:** Results of image alignment by each method.

Method	Number of Matched Points for Image Alignment	$\mathrm{Mean}\;\mathrm{of}\;R_{rot}/R_{ref}\pm \left(Standard\;Deviation\right)$
DoG detector	443	I: 1.010±0.043 II: 1.012±0.039 III: 1.010±0.045
Hessian corner detector	60	I: 1.011±0.044 II: 0.980±0.150 III: 1.011±0.065
Proposed method	157	I: 1.010±0.043 II: 1.012±0.039 III: 1.010±0.047
Black marker	—	I: 1.010±0.043 II: 1.010±0.044 III: 1.012±0.039

## Data Availability

Data sharing not applicable.
